# Adiponectin and Sarcopenia: A Systematic Review With Meta-Analysis

**DOI:** 10.3389/fendo.2021.576619

**Published:** 2021-04-15

**Authors:** Klara Komici, Antonio Dello Iacono, Antonio De Luca, Fabio Perrotta, Leonardo Bencivenga, Giuseppe Rengo, Aldo Rocca, Germano Guerra

**Affiliations:** ^1^ Department of Medicine and Health Sciences “Vincenzo Tiberio”, University of Molise, Campobasso, Italy; ^2^ School of Health and Life Sciences, University of the West of Scotland, Hamilton, United Kingdom; ^3^ Section of Human Anatomy, Department of Mental and Physical Health and Preventive Medicine, University of Campania “Luigi Vanvitelli”, Naples, Italy; ^4^ Department of Translational Medical Sciences, University of Naples “Federico II”, Naples, Italy; ^5^ Department of Advanced Biomedical Sciences, University of Naples “Federico II”, Naples, Italy; ^6^ Istituti Clinici Scientifici Maugeri SpA Società Benefit (ICS Maugeri SpA SB), Telese Terme, Italy

**Keywords:** adiponectin, sarcopenia, aging, elderly, muscle mass

## Abstract

**Background:**

Sarcopenia is a progressive loss of skeletal muscle mass whose pathophysiology has been proposed to possibly involve mechanisms of altered inflammatory status and endocrine function. Adiponectin has been shown to modulate inflammatory status and muscle metabolism. However, the possible association between adiponectin levels and sarcopenia is poorly understood. In order to fill this gap, in the present manuscript we aimed to summarize the current evidence with a systematic review and a meta-analysis of studies reporting serum adiponectin levels in patients with sarcopenia compared to non-sarcopenic controls.

**Methods:**

An electronic search through Medline/PubMed, Cochrane Library, and Science Direct was performed till March 1, 2020. From the included papers, meta-analysis of cross-sectional studies comparing serum levels of adiponectin between patients with sarcopenia and controls was performed.

**Results:**

Out of 1,370 initial studies, seven studies were meta-analyzed. Sarcopenic participants had significantly higher levels of adiponectin Hedges’ g with 95% confidence interval (CI): 1.20 (0.19–2.22), p = 0.02 than controls. Subgroup analysis, performed in Asian population and focused on identification of the condition based on AWGS criteria, reported higher adiponectin levels in sarcopenic population (2.1 (0.17–4.03), p = 0.03 and I2 = 98.98%. Meta-regression analysis revealed female gender to significantly influence the results as demonstrated by beta = 0.14 (95% CI (0.010–0.280), p = 0.040).

**Conclusions:**

Our meta-analysis found evidence that sarcopenia is associated with higher adiponectin levels. However, caution is warranted on the interpretation of these findings, and future longitudinal research is required to disentangle and better understand the topic.

## Introduction

Sarcopenia is a disease of massive concern in clinical epidemiology as a result of its rising prevalence inherently associated with the aging trends and the increasing elderly populations all over the world. It is common in men and women, with a prevalence of about 10% in those aged >65 years. Sarcopenia is clinically defined as a progressive and generalized musculoskeletal disease associated with increased likelihood of adverse health and functional outcomes including falls, fractures, physical disability and mortality (1). A few factors are recognized as possible pathophysiological mechanisms of sarcopenia and concurrent muscle loss, including among others neuromuscular aging, malnutrition, insufficient physical activity, alternations of inflammatory status and endocrine function (2, 3). The degenerative effects of malnutrition and insufficient physical activity on the neuromuscular function and functional capacity of the musculoskeletal system during aging have been object of extensive investigations, and are routinely acted upon to preserve muscle function, muscular strength, and muscle power for the health and wellbeing of older adults. Conversely, less and inconclusive evidence is available to elucidate the mediating role endocrine and inflammation homeostases have for the onset and degeneration of sarcopenia. Adiponectin is the most abundantly expressed human body adipokine, predominantly produced by bone marrow adipose tissue, that mediates inflammatory processes by inhibiting the synthesis of IL-6, IL-18, TNF-α, and blocking the activation NF-κB (4, 5). Furthermore, adiponectin plays a protective role in cardiovascular health by inhibiting foam cell formation and adhesion molecule expression (6) and facilitating the systemic insulin sensitivity (7). Recent studies have revealed that adiponectin signaling activation may have a protective role against muscle atrophy through binding to T-cadherin and consequently promoting muscle regeneration (8, 9). Importantly, the beneficial effects of physical exercise on muscle metabolism are also mediated *via* adiponectin signaling (10). In view of the effective role of physical activity in the prevention and treatment of sarcopenia (11, 12), clarifying the underpinning mechanisms in which adiponectin signaling concurs seems prudent. In fact, previous studies examining adiponectin circulating levels in sarcopenic population have reported contrasting results (13). This may be due to different clinical settings, population characteristics or, different tools used for sarcopenia definition.

Diagnosis and monitoring of sarcopenia is based on clinical, functional, and imaging evaluations. Muscle mass imaging assessments using magnetic resonance imaging (MRI), computed tomography (CT), or dual-energy x-ray absorptiometry (DXA) scan are validated methods for assessing muscle mass, but they are burdened by intrinsic limitations and high cost that confine their use to research activity rather than clinical practice. Physical performance assessment consists of standardized and low-cost tests, with relevant ability to predict the development of disability. Nevertheless, in clinical practice, their application may be altered by specific conditions or comorbidities, whose prevalence is higher in elderly (14).

Circulating adiponectin level measurements only require collection of blood samples and laboratory techniques, as enzyme linked immunosorbent assay (ELISA), which represents a valid widespread and low-cost method. It has been reported that circulating adiponectin levels are negatively associated with physical performance in adult population (15). Accordingly, exercise training and dietary interventions have been shown to modulate adiponectin levels, as emerged by cross-sectional studies and randomized controlled trials reporting that aerobic and resistance exercise training may influence the adipokine levels (16–18). Low protein intake enhanced adiponectin expression in experimental models (19); of note, a clinical trial focused on the effects of low caloric diet with different protein contents on adiponectin profile, showing that 35% protein diet regimen significantly modified adiponectin levels (20). Lifestyle interventions as resistance exercise training and adequate nutritional intake are the current approaches for the management and treatment of sarcopenia. In this context, adiponectin would be helpful to follow sarcopenia progression, also allowing to monitor response to treatment.

Therefore, we conducted a systematic review and meta-analysis of the studies that compared adiponectin levels between sarcopenic and non-sarcopenic subjects as to evaluate the association between adiponectin level and sarcopenia. In addition, we investigated whether this association is influenced by sarcopenia definition, tools used for its diagnosis, or population characteristics.

## Materials and Methods

This systematic review was performed according to the Strengthening the Reporting of Observational Studies in Epidemiology (STROBE) (21) criteria and the recommendations in the Preferred Reporting Items for Systematic Reviews and Meta-Analyses (PRISMA) statement (22). The protocol has been registered in Prospero database (registration ID: CRD42020176530).

### Data Sources and Searches

Two reviewers performed a systematic search from date of inception to 1 of march 2020 using four electronic databases: Medline/PubMed, Cochrane Library and Science Direct. Potentially relevant studies were subsequently screened by full text reading, independently by two reviewers. A third reviewer resolved any possible disagreement by discussion and reaching a consensus. Combination of the following free text terms and major medical subject headings adapted to the requirements of each database were used: “adiponectin,” “AdipoQ,” “ACRP30,” “sarcopenia,” “muscle mass.” Boolean Logical Operators AND, OR were used. As an example Medline/PubMed search: (“adiponectin”[MeSH Terms] OR “adiponectin”[All Fields] OR “adiponectin s”[All Fields] OR “adiponectine”[All Fields] OR “adiponectins”[All Fields] OR “AdipoQ”[All Fields] OR “ACRP30”[All Fields]) AND (“sarcopenia”[MeSH Terms] OR “sarcopenia”[All Fields]); (“adiponectin”[MeSH Terms] OR “adiponectin”[All Fields] OR “adiponectin s”[All Fields] OR “adiponectine”[All Fields] OR “adiponectins”[All Fields] OR “AdipoQ”[All Fields] OR “ACRP30”[All Fields]) AND ((“muscle s”[All Fields] OR “muscles”[MeSH Terms] OR “muscles”[All Fields] OR “muscle”[All Fields]) AND (“molecular weight”[MeSH Terms] OR (“molecular”[All Fields] AND “weight”[All Fields]) OR “molecular weight”[All Fields] OR “mass”[All Fields])) An additional reviewer was involved in the selection process, with particular task of solving conflicting opinions cases. Reference lists of retrieved papers and review articles were hand-searched for additional relevant citations. Only peer-reviewed articles published in English were included in the meta-analysis.

### Study Selection

The studies initially obtained using the search strategy were assessed for eligibility by screening titles and abstracts, independently by two authors. Potentially relevant studies were subsequently screened by full text reading, independently by two reviewers and a third reviewer, which also resolved any possible disagreement by discussion and reaching a consensus. Studies included in the systematic review met the following inclusion criteria: a) diagnosis of sarcopenia based on European Working Group on Sarcopenia in Older People (EWGSOP), Asian Working Group for Sarcopenia (AWGS) (1, 23), consensuses, or any other definition used by original studies’ author; b) serum or plasmatic levels of adiponectin reported; c) included a control group (non-sarcopenic population). Studies were excluded if: a) not clear diagnosis of sarcopenia was reported; b) animal or *in vitro* studies; c) no report or measure of quantitative adiponectin levels was reported.

### Data Extraction and Quality Assessment

Two reviewers independently extracted the following variables from the included studies: first author’s name, year of publication, total number of individuals included in the study, study design, mean or median age of individuals, mean or median Body Mass Index (BMI), percentage of males population, continent, applied definition(s) of sarcopenia, method used for sarcopenia identification, mean muscle mass and physical performance in sarcopenia and non-sarcopenia group, mean or median adiponectin levels in sarcopenia and non-sarcopenia population. Risk of bias of the included studies was assessed using the Newcastle Ottawa Scale (NOS) (24) for case–control and cohort studies and a modified version of the NOS for cross- sectional studies. A system of points was given to the eligible categories: (I) selection of the study population, (II) comparability, and (III) description of the outcome. A study was given a maximum of one point in each item within the Selection and Outcome categories and a maximum of two points was given for the Comparability category. The scale scores varied depending on the study design. For case–control and cohort studies, it ranged from 0 to 9 points with ≥7 points classified as high quality. For cross-sectional studies, it ranged from 0 to 7 points. A median of ≥4 points was considered as high quality for cross-sectional studies as described by other authors (25, 26).

### Statistical Analysis

Analyses were performed using STATA SE 16.1. Adiponectin level was meta-analyzed when at least three studies contributed data. Hedges’ g was used for standardized mean differences calculation. Calculations of mean and standard deviation (SD) for different groups were performed according to Cochrane Handbook for Systematic Reviews of Interventions (27). As necessary estimation of mean and SD was calculated from sample size and inter quartile range (IQR) (28). Study heterogeneity was measured using the chi2 and I2 statistics (29). I2 values greater than 25% were considered to reflect low heterogeneity, 50% moderate, and 75% high heterogeneity. A random statistical analysis model was used. Given significant heterogeneity, a meta-regression analysis was performed using differences in mean age, BMI and percentage of female population as moderators. Moreover, subgroup analysis was conducted considering diagnosis of sarcopenia according to the definition of AWGS, and diagnostic methods used for sarcopenia confirmation such as (DXA) or Bioelectrical Impedance Analysis (BIA). Forest plots were used to visualize the results. Funnel plots were used initially to evaluate visually publication bias while Egger’s regression test and Begg’s test were used to inferentially evaluate publication bias (30, 31).

## Results

### Search Results

**Figure 1:**
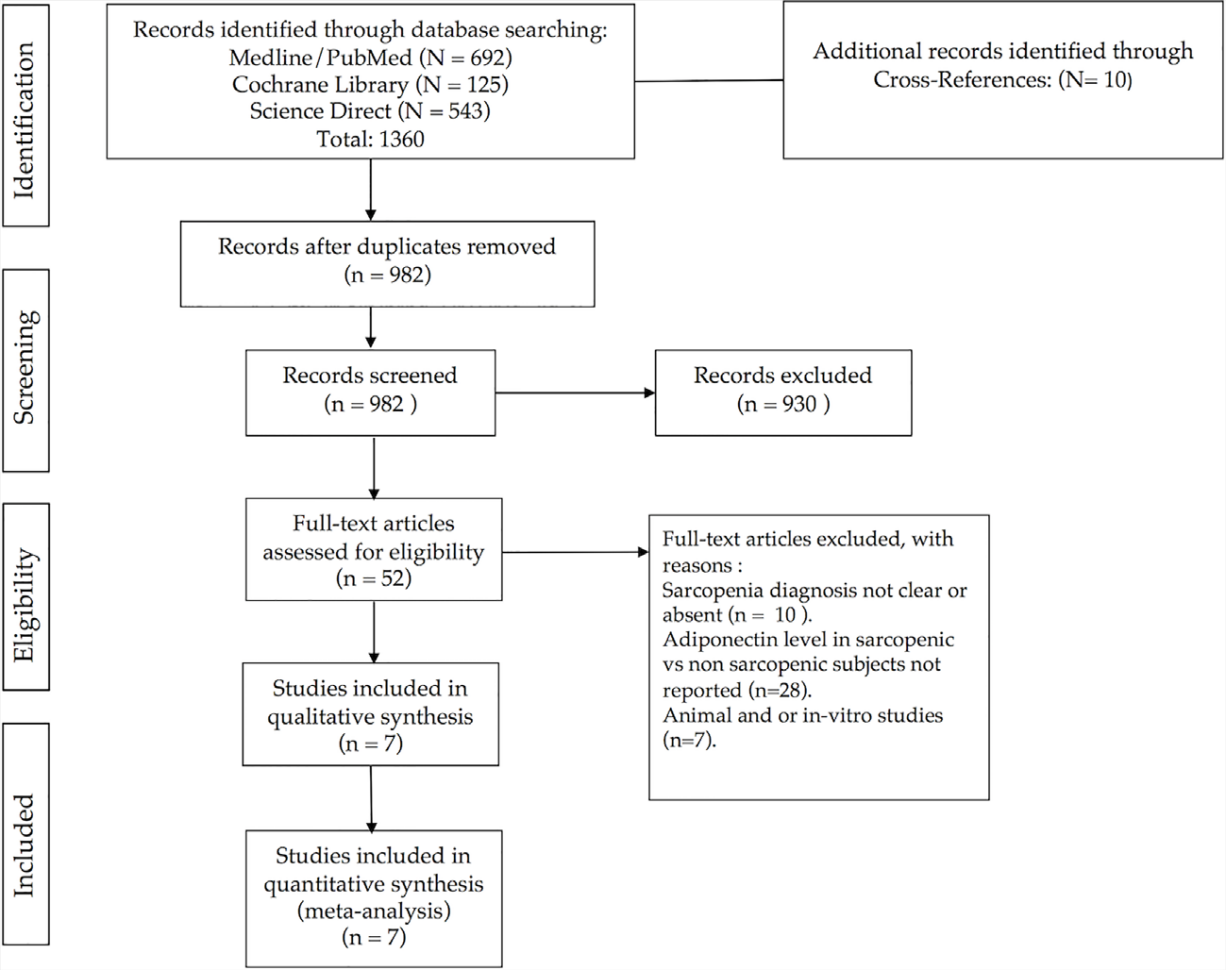
Flowchart of study selection.

A total of 1370 studies were retrieved through electronic database searches and cross-references search. After removal of duplicates, 982 studies were identified for title and abstract screening. Review of the titles and abstracts yielded 52 relevant studies for full text screening. From these, seven studies were conducted on animal or *in vitro* models, 28 studies did not report quantitative expression of adiponectin level in sarcopenic and non-sarcopenic subjects, and in other 10 studies the reported diagnostic criteria for sarcopenia were not clear or absent. Finally, we included seven studies in the meta-analysis. **Figure 1** shows the study flow-chart and searching results. **Supplemental Table 2**, reports the 982 screened articles with details regarding full-text reading or only title or abstract screening.

### Study Characteristics

Study characteristics are described in **Table 1**. A total of 1,389 individuals, with a mean age ranging from 40.3 to 80. 2 years were included, and sample size ranged from 77 to 539 individuals. Six studies presented cross-sectional design (32–34, 36–38), while one study reported retrospective data (35). Five studies included community-dwelling individuals, while one study hospitalized patients prior to cardiac surgery and one study included data from both hospitalized and geriatric outpatients. Four studies used DXA to identify sarcopenia while two studies identified sarcopenia through BIA. The most common definition of sarcopenia was AWGS. Included studies reported serum measurements of adiponectin through ELISA or radioimmunoassay.

**Table 1:** Main characteristics of studies included in the meta-analysis.

First Author and Year	Study Design	Setting	Population	Sarcopenia Definition	Diagnostic Tools	Muscle Mass in Sarcopenic Population	Handgrip strength in Sarcopenic Population	Adiponectin Measurement	Study Main Findings
Ramachandran et al. (32)	Cross Sectional	Community Dwelling	*n= 539* M: n=280 F: n= 259	The lowest sex-specific tertile: 93.8 cm^2^ in women and 110.7 cm^2^ in men) of adjusted thigh muscle area.	CT scan of the thigh.	91.08 ± 7.66 cm^2^	NA	Radio-immunoassay	Elderly subjects with central and global adiposity are more likely to have worse glucose tolerance. A strong negative association between circulating adiponectin levels and glucose disposal rates after adjustment for confounders is described.
Kim et al. (33)	Cross Sectional	Community Dwelling	*n=288* *M: n=171* *F: n=117*	ASM/weight (%) of 1 SD below the sex-specific mean value for the young reference group. Cutoff point for sarcopenia: 30.9% in men and 24.5% in women.	DXA	17.53 ± 4.9	NA	ELISA	Serum adipocyte fatty binding acid protein levels are higher in sarcopenia vs non sarcopenia, both men and women
Can et al. (34)	Cross Sectional	Community Dwelling	*n* = 77 M: *n* = 27 F: *n* = 45	EWGSOP	BIA	18.4 ± 4.0 kg	14.2 ± 7.1 kg	ELISA	Sarcopenia is associated with higher levels of CRP, ESR and lower levels of adiponectin
Harada et al. (35)	Retrospective	In hospital patients prior to cardiac surgery	*n* = 132 M: *n* = 80 F: *n* = 52	AWGS	BIA	5.20 ± 0.71 kg/m^2^	12.6 ± 5.9 kg	ELISA	Adiponectin and sialic acid were significantly higher in sarcopenic than non-sarcopenic CVD patients.
Lu et al. (36)	Cross Sectional	Community Dwelling	*n* = 189 M: *n* = 70 F: *n* = *119*	AWGS	DXA	5.86 ± 1.04 kg/m^2^	NR	ELISA	Sarcopenic elderly showed lower BMI and leptin and higher adiponectin and high-density lipoproteins. Levels of EAA, branched-chain AAs and choline, were inversely associated with sarcopenia.
Rossi et al. (37)	Cross Sectional	Community Dwelling	*n* = 57 M: *n* = NR F: *n* = NR	NHANES	DXA	20.2 ± 3.9 kg	23.9 ± 6.2 kg	Serum ELISA	Sarcopenic individuals showed greater adiponectin concentration adiponectin/fat mass ratio adiponectin/visceral and higher PAI-1.
Li et al. (38)	Cross Sectional	Community, Nursery Care and in hospital patients	*n* = 112 M: *n* = 50 F: *n* = *62*	AWGS	DXA	5.78 ± 0.78 kg/m^2^	23.2 ± 7.34 kg	ELISA	High levels of the inflammatory cytokines TWEAK and TNF-α are associated with an increased risk of sarcopenia, while IGF-1, insulin, and adiponectin are associated with a decreased risk of sarcopenia

AWGS, Asian Working Group for Sarcopenia; BIA, bioelectrical impedance analysis; CVD, cardio-vascular disease; DXA, dual-energy X ray absorptiometry; EWGSOP, European Working Group on Sarcopenia in Older People; ELISA, enzyme-linked immunosorbent assay; ESR, erythrocyte sediment ratio; EAA, plasma essential amino acids; IGF-1, insulin growth factor-1; TNF-alfa, tumor necrosis factor alfa; TWEAK, TNF-like weak inducer of apoptosis; PAI-1, plasminogen activator inhibitor-1.

### Adiponectin Expression and Meta-analysis Findings

**Figure 2:**
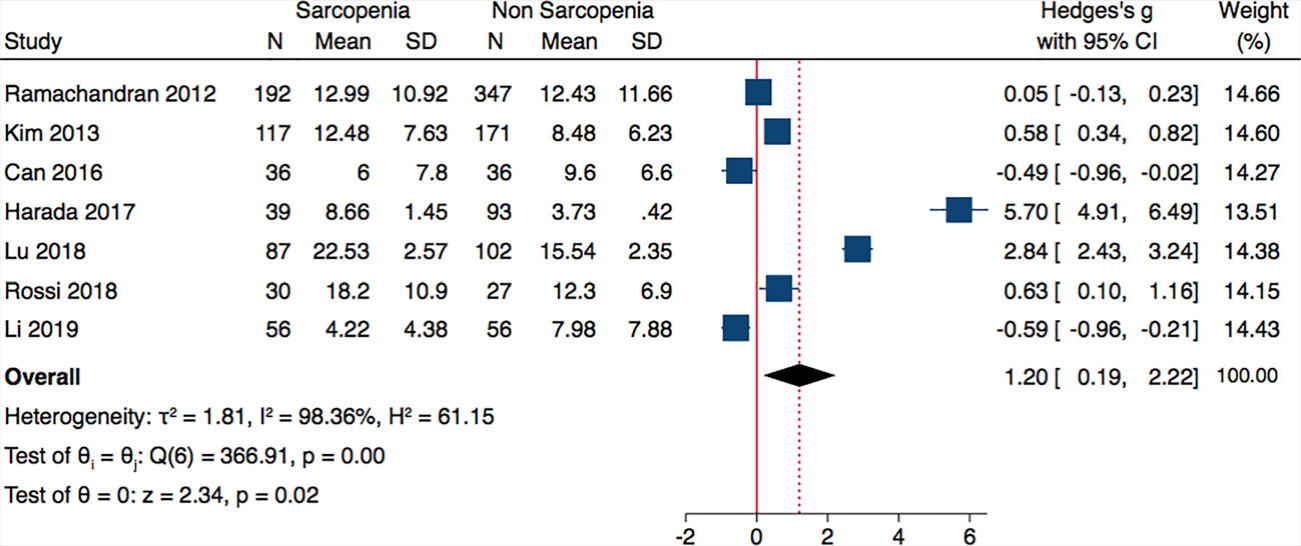
Forest plot of serum adiponectin levels in sarcopenic vs. no sarcopenic subjects. SD, standard deviation; CI, confidence interval.

Meta-analysis of seven included studies, revealed that individuals with sarcopenia (n= 557), compared to individuals without sarcopenia (n=832), were more likely to have significantly higher adiponectin levels: (Hedges’ g with 95% confidence interval [CI], 1.20; 0.19–2.22; p = 0.02). The studies were characterized by high heterogeneity (I^2^ = 98.36%, p < 0.0001) (**Figure 2**).

**Figure 3:**
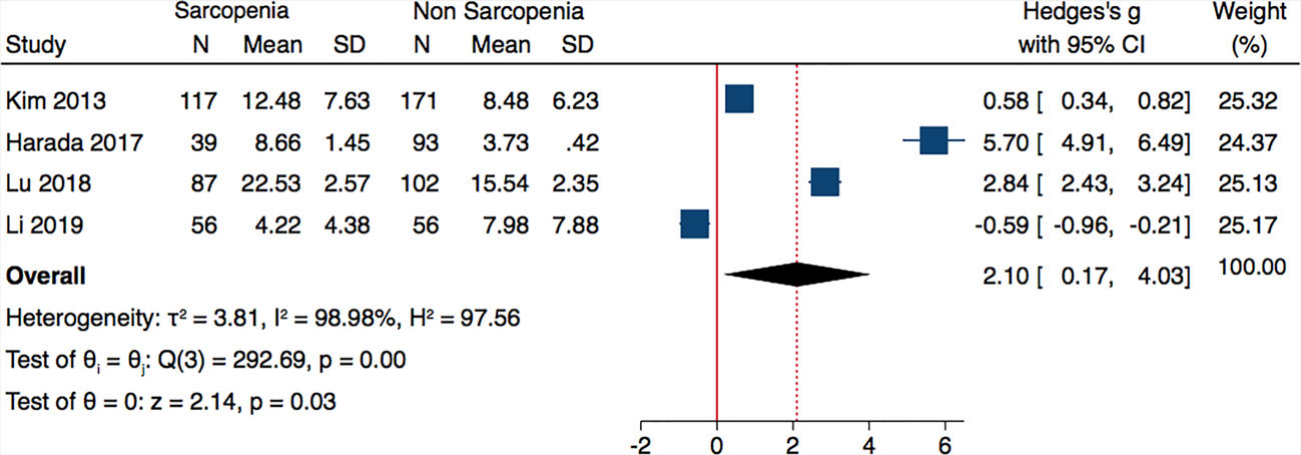
Forest plot of serum adiponectin levels in sarcopenic vs. no sarcopenic subjects. Studies performed on Asian population and or based on AWGS criteria. SD, standard deviation; CI, confidence interval.

**Figure 4:**
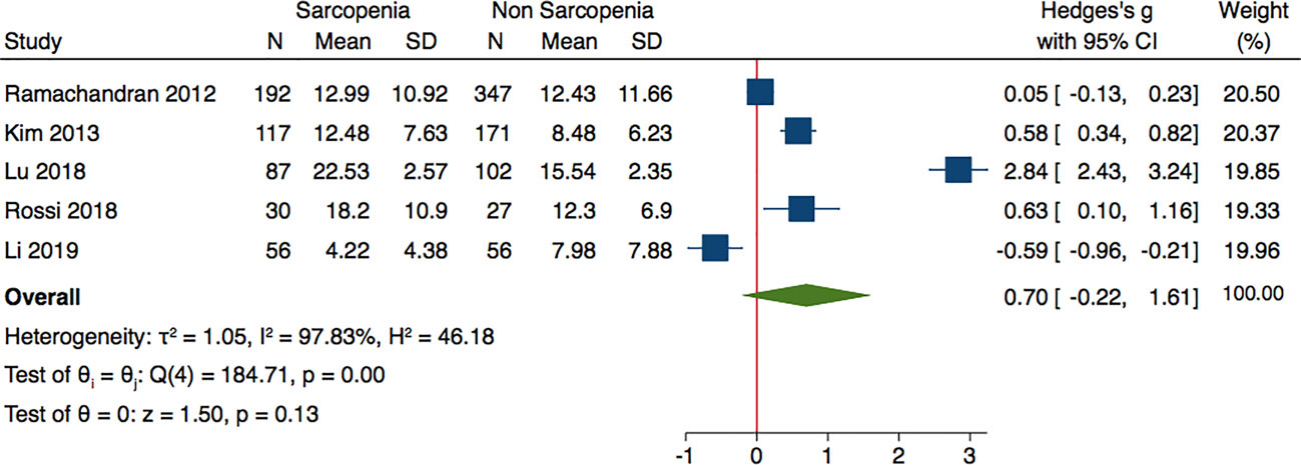
Forest plot of serum adiponectin levels in sarcopenic vs. no sarcopenic subjects. Studies applying DXA. SD, standard deviation; CI, confidence interval.

Analyzing the data of four studies performed in Asian population and following AWGS criteria, sarcopenic individuals were characterized by higher levels of adiponectin: (Hedges’ g with 2.1; 0.17–4.03; p = 0.03 and I^2^ = 98.98%) (**Figure 3**). The subgroup analysis of studies based on DXA evaluation of sarcopenia, did not report significantly different adiponectin levels (Hedges’ g with 95% CI, 0.70; −0.22 to 1.61; p = 0.13; I^2^ = 97.83) (**Figure 4**).

### Meta-Regression Analysis

Since the differences in adiponectin levels between sarcopenic and non-sarcopenic population were characterized by a high heterogeneity (I2 ≥ 50%, p < 0.05), we ran a meta-regression analysis to seek potential moderators, such as age, BMI differences and percentage of female population. While age and BMI did not affect the findings (beta = 0.11; 95% CI, −1.40 to 1.63; p = 0.842; beta = 0.85; 95% CI, −1.76 to 3.47; p =0.416), respectively, gender was found to moderate the results (beta = 0.14; 95% CI, 0.010–0.280; p=0.040).

### Study Quality and Publication Bias

The quality of studies evaluated by NOS was good. Results of the NOS quality assessment of the included studies are reported in **Supplemental Material Table 1**. Asymmetry was observed by visual inspection of funnel plots (**Supplemental Figures 1**–**3**). However, Egger’s regression test (p = 0.133) for all studies (p = 0.122), for AWGS and Asian population studies, and (p = 0.652) for studies using DEXA indicated no significant publication bias among the studies in this meta-analysis.

## Discussion

From the overall meta-analysis on 1,389 individuals, 557 sarcopenic and 832 controls, it emerged that sarcopenic individuals are characterized by significantly higher adiponectin levels. The difference regarding the prevalence of females in sarcopenia and non-sarcopenia population resulted as a significant moderator and may explain, at least in part the heterogeneity found in our results. It should be mentioned that the included studies did not always report the same relationship between sarcopenia and adiponectin, and different results were described even in studies following AWGS criteria and performed on Asian population or using DEXA as a diagnostic method. Many studies emphasize the role of adiponectin on skeletal muscle metabolism and function (39–42). In vitro studies performed on primary human and animal skeletal muscle cells reported that adiponectin increased glucose up-take in muscle cells and this action is mediated by the translocation of the glucose transporter GLUT4 to the cell surface (43). Furthermore, adiponectin stimulates the interaction of adaptor protein containing pleckstrin homology domain (APPL1) with adiponectin receptors AdipoR1 and AdipoR2, leading to increased GLUT4 membrane translocation, and knock down of APPL1 resulted in significant reduction of glucose uptake and insulin sensitivity (44). At least in young individuals, insulin inhibits protein catabolism in muscle (45), and muscle accretion is mediated through activation of p38 MAPK signaling. The delivery of amino acids is related to enhanced perfusion which is impaired in aged muscle (46). With aging, insulin sensitivity decreases, whereas preservation of glucose homeostasis and insulin sensitivity is associated to longevity (47). Moreover, mice with fat-specific disruption of the insulin receptor gene (FIRKO) are characterized by lower fasting insulin levels, elevated serum adiponectin levels and enhanced longevity (48). Human studies including centenarians, report increased levels of adiponectin and inverse correlation with homeostasis model assessment for insulin resistance (HOMA-IR) or inflammatory markers (49, 50).

On the other hand, age- related reduction of protein metabolism and reduction of insulin mediated suppression of proteolysis leads to development of anabolic resistance and sarcopenia. Of interest, reduced adiponectin levels has been associated to activation of muscle protein degradation as ubiquitin-proteasome proteolytic pathway mediated by insulin resistance (51).

In contrasts, studies focused on chronic heart failure patients, reveal that high adiponectin levels are associated to HF severity and increased mortality (52). A recent meta-analysis also reported that high adiponectin levels increased the risk for both cardiovascular and all cause mortality, but this effect was attenuated by adjustment for natriuretic peptides levels (53).

Inflammation is considered another key factor in the genesis of sarcopenia (3). The mechanisms underpinning sarcopenia and chronic inflammation include NF-kB and TNF-alfa activation as primary mediators and principal pathway, and are characterized by increased muscle protein imbalance, alternation of mitochondrial function, cell death or apoptosis, and restricted muscle repair and regeneration (54). In animal models of low inflammation grade, an inverse correlation was found between cytokin levels and muscle wasting degree (55). Increased circulating levels of IL-1, IL-6, CRP, and tumor necrosis factor-alpha (TNF-alfa) are described in the elderly (56). Furthermore, a recent meta-analysis study reported that sarcopenic subjects are characterized by significantly elevated inflammatory markers (57). The cross-talk between adiponectin and inflammatory signaling is discussed in abundance. It has been suggested that adiponectin attenuates the inflammatory responses to multiple stimuli by modulating signaling pathways in a variety of cells (58). In experimental models of caloric restricted and aged animals, it has been reported a reduced secretion of IL-6, increased insulin sensitivity, amelioration of physical performance and at least in part these modifications are related to enhanced expression and secretion of adiponectin (59). Restoring adiponectin levels *via* modification of gene expression resulted in reduced TNFα, IL-1β, and CD68 levels, greater expression regeneration proteins like myogenin, Myh3, Myh7, and larger muscle fibers (60).

In our meta-analysis study, increased levels of adiponectin were associated with sarcopenia. Li et al. (38) reported that lower adiponectin levels were associated with high sarcopenia risk, but a 12-week intensive lifestyle intervention program led to significant improvements in muscle mass, muscle strength, and gait speed. Moreover, adiponectin levels considerably increased post-intervention, even above the levels registered in non-sarcopenic elderly subjects. Similar results regarding adiponectin levels between sarcopenic and non-sarcopenic population was reported also by Can et al. (34). However, the studies Lu et al. (36), Rossi et al. (37), Kim (32), and Harada (33) found opposite results.

Although, increased adiponectin levels in sarcopenia appears to be controversial, some possible mechanisms have been proposed to explain the detected peculiarity: a) down-regulation of adiponectin receptor signaling (61) b) deposition of adipose tissue in muscles that may influence adiponectin expression (62) c) activation of catabolism related to the presence of other comorbidities (63).

Discrepancies in the current literature may also be dependent on the confounding effect of adiponectin resistance and the resultant rise of adiponectin with ageing. However, in our meta-regression analysis, age difference between sarcopenic and non-sarcopenic populations was not a significant moderator of the results. It is worth mentioning that in some of these studies, population was heterogenous regarding gender, and meta-regression analysis found that the difference in female gender prevalence between sarcopenic and non-sarcopenic groups was a significant moderator, which in turn may at least in part explain the high heterogeneity rescued. In the study by Harada et al. (33), among non-sarcopenic population female prevalence was about 28%, whereas in sarcopenic population around 66.7%. This was a retrospective study performed in a cardiovascular setting, including patients undergoing cardiovascular surgery, and the later presentation of cardiovascular diseases and the higher comorbidity burden among females may explain these differences (64). While Li et al. (36), in a cross-sectional study reported that the sarcopenic group is characterized by higher age, more female and higher percentage of diabetic subjects. It has also been reported that the prevalence of sarcopenia differs between men and women (65).

We could not perform addition analysis regarding cardiovascular risk factor such as diabetes or dyslipidemia that could modify the results as these data were not available. In addition, subgroup analysis, performed only on Asian population diagnosed according to AWGS criteria, also reported significantly higher adiponectin levels in sarcopenic population. Subgroup analysis of studies using DXA failed to report any significant difference regarding adiponectin levels; however, this may be explained by different populations characterizing the included studies. We could not perform any additional subgroup analysis since the number of studies performed in U.S or Europe were not adequate to perform a meta-analysis.

In a study performed on community dwelling, older adults were found to have a significant and independent association between circulating adiponectin and frailty status: higher adiponectin levels in frail elderly than in their non-frail counterparts (66). It should be reminded that poor physical functioning, weight loss, and low muscle mass are the crucial components of frailty in older adults. Baker et al. (67) also uncovered a significant relationship between high adiponectin and weight loss, low muscle mass, and low muscle density. Furthermore, in this study, adiponectin was substantially associated with increased risk of incident disability and all-cause mortality. These findings are in agreement with the results of our meta-analysis, which points to sarcopenia and the associated catabolic stress as the pathophysiological background causing upregulation of adiponectin as part of compensatory mechanisms against inflammation and oxidative stress (68).

Moreover, our meta-regression analysis suggests an important role of female gender in explaining the association between sarcopenia and adiponectin levels. This finding seems to be pertinent with the current literature, suggesting that women present higher adiponectin levels than men. Indeed, women express greater plasma adiponectin than men, independent of fat mass and BMI, and there has been suggestion that this relationship is in part influenced by sex hormones (69, 70).

In addition to general obesity, abdominal obesity has been reported as an independent predictor of lower circulating adiponectin levels (71, 72). Furthermore, higher adiponectin levels have been described in obese individuals with higher subcutaneous to visceral adipose tissue ratio, thus allowing to speculate on the role of adipose tissue distribution in adipokine release (73). Indeed, adipose tissue sampling in individuals undergoing abdominal elective surgery reported decreased adiponectin secretion from central adipose tissue, while subcutaneous adipose tissue secretion remained unchanged (74).

From our meta-regression analysis, BMI did not significantly influence the results, and additional data as waist circumference or trunk fat could not be included. However, upregulation of adiponectin expression in muscles of obese and diabetic mice has been previously observed (75); the assessment of metabolic or inflammatory profile of subjects suffering from low muscle mass and impaired physical function revealed a more significant association with insulin resistance and elevated levels of C-reactive protein, IL-1, and IL-6 in obese patients than in subjects with normal or reduced BMI (76, 77). It is plausible that sarcopenic obesity is characterized by distinct metabolic and inflammatory profiles and adiponectin expression in this category deserves further investigation.

Considering that the current meta-analysis is the first of this kind, our findings should be interpreted with caution for a few reasons. First and utmost, we observed high heterogeneity in all the cross-sectional analyses conducted. Nevertheless, the Meta-analysis of Observational Studies in Epidemiology (MOOSE) guidelines state that when analyzing observational data, heterogeneity is to be expected. Second, we were not able to investigate the effects of physical exercise or nutritional intervention strategies on adiponectin levels and the indirect impact on outcome measures in sarcopenic populations. However, previous meta-analyses reported significant modifications of adiponectin levels in obese or diabetic population following physical exercise intervention, which presume similar effects also among sarcopenic people (12, 78). Thus, future longitudinal research should seek to investigate if adiponectin could be a potential biomarker of sarcopenia identification and prediction.

## Conclusions

In conclusion, our meta-analysis found evidence that sarcopenia is associated with higher adiponectin levels. However, caution on the interpretation of these findings are warranted and future longitudinal research is required to disentangle and better understand the relationships that emerged.

## Data Availability Statement

The raw data supporting the conclusions of this article will be made available by the authors, without undue reservation.

## Author Contributions

Conceptualization, KK and GG. Methodology, KK and AI. Software, KK and FP. Validation, AL, AR, and LB. Formal analysis, KK, AR, and LB. Investigation, KK and AI. Resources, KK and GG. Data curation, KK, FP, GG, and AR. Writing original draft preparation, AI, KK, and GG. Supervision, GG and GR. All authors contributed to the article and approved the submitted version.

## Conflict of Interest

The authors declare that the research was conducted in the absence of any commercial or financial relationships that could be construed as a potential conflict of interest.

## Supplementary Material

Supplementary Table 1 NOS quality assessment of the included study.

Supplementary Figure 1 Funnel plot of serum adiponectin levels in sarcopenic vs. no sarcopenic subjects.

Supplementary Figure 2 Funnel plot of serum adiponectin levels in sarcopenic vs. no sarcopenic subjects. Studies performed on Asian population and or based on AWGS criteria.

Supplementary Figure 3 Funnel plot of serum adiponectin levels in sarcopenic vs. no sarcopenic subjects. Studies applying DXA.
