# Supplementary material for: Adiponectin and Sarcopenia: A Systematic Review With Meta-Analysis
Source: Front Endocrinol (Lausanne). 2021 Apr 15;12:576619. doi: 10.3389/fendo.2021.576619 (PMC8082154; doi:10.3389/fendo.2021.576619)
Supplement: Supplementary Table 1 — NOS quality assessment of the included study. [file Table_1.docx]

|  | Li et al 2019 | Rossi et al 2018 | Lu et al 2018 | Harada et al 2017 | Can et al 2016 | Kim et al 2013 | Ramachandran  et al 2012 |
| --- | --- | --- | --- | --- | --- | --- | --- |
| Representativeness of the exposed cohort | **🟑** | **🟑** | **🟑** | **🟑** | **🟑** | **🟑** | **🟑** |
| Selection of the non exposed cohort | **🟑** | **🟑** | **🟑** | **🟑** | **🟑** | **🟑** | **🟑** |
| Ascertainment of exposure | **🟑🟑** | **🟑🟑** | **🟑🟑** | **🟑🟑** | **🟑🟑** | **🟑🟑** | **🟑🟑** |
| Demonstration that outcome of interest was not present at start of study | **🟑** | **🟑** | **🟑** | **🟑** | **🟑** |  |  |
| Comparability of cohorts on the basis of the design or analysis | **🟑🟑** | **🟑** | **🟑** | **🟑** | **🟑** | **🟑🟑** | **🟑🟑** |
| Assessment of outcome | NA | NA | NA | NA | NA | NA | NA |
| Was follow-up long enough for outcomes to occur | NA | NA | NA | NA | NA | NA | NA |
| Adequacy of follow up of cohorts | NA | NA | NA | NA | NA | NA | NA |
| Total | 7 | 6 | 6 | 6 | 6 | 7 | 7 |
