# Supplementary figures and images for: Adiponectin and Sarcopenia: A Systematic Review With Meta-Analysis
Source: Front Endocrinol (Lausanne). 2021 Apr 15;12:576619. doi: 10.3389/fendo.2021.576619 (PMC8082154; doi:10.3389/fendo.2021.576619)

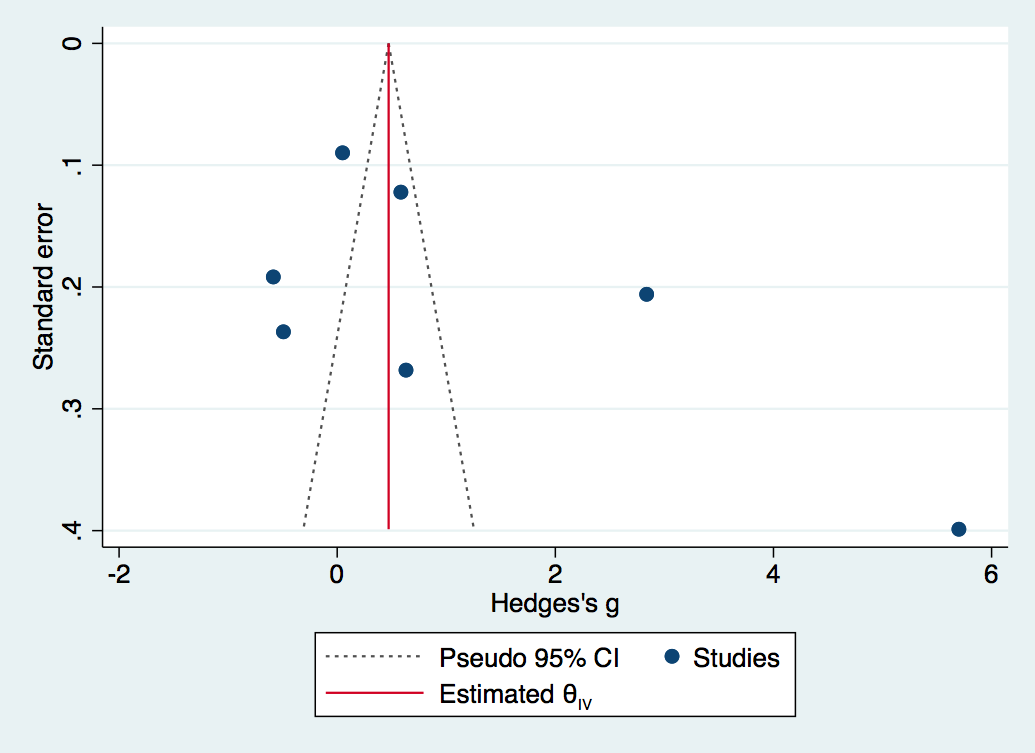

Supplement: Supplementary Figure 1 — Funnel plot of serum adiponectin levels in sarcopenic vs. no sarcopenic subjects. [file Image_1.tif]

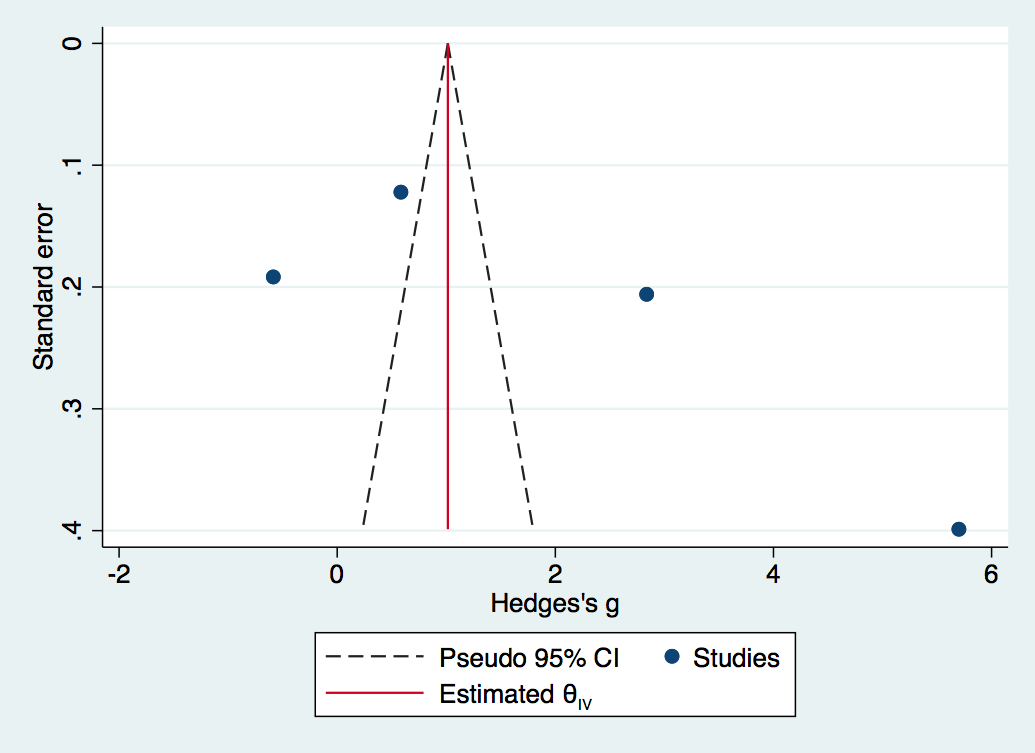

Supplement: Supplementary Figure 2 — Funnel plot of serum adiponectin levels in sarcopenic vs. no sarcopenic subjects. Studies performed on Asian population and or based on AWGS criteria. [file Image_2.tif]

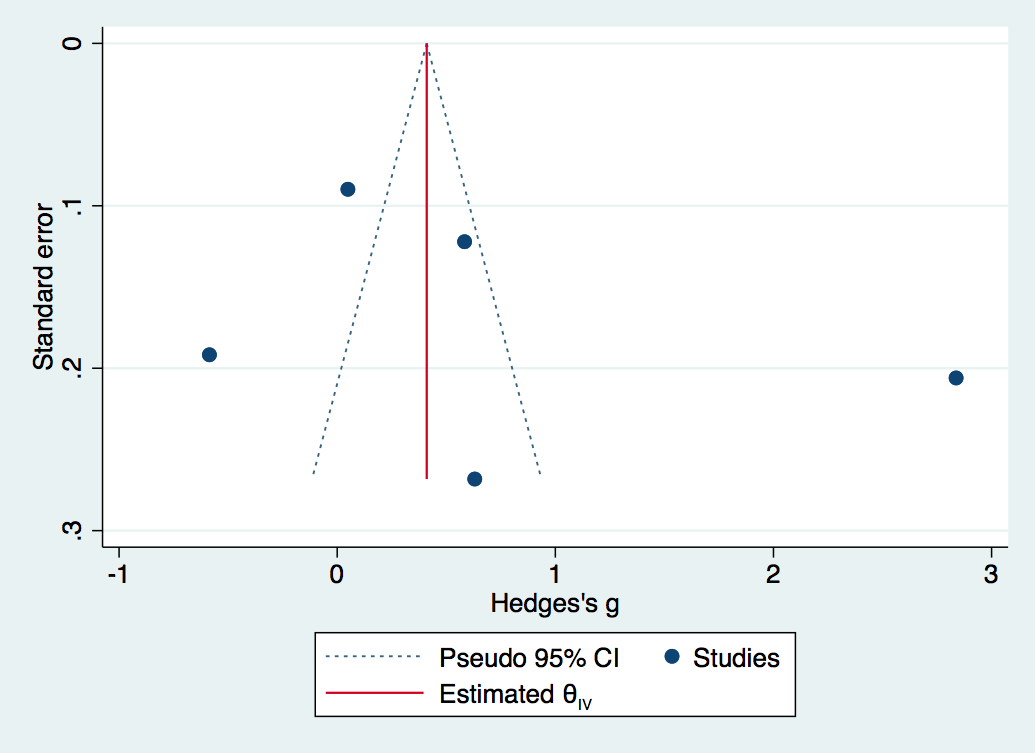

Supplement: Supplementary Figure 3 — Funnel plot of serum adiponectin levels in sarcopenic vs. no sarcopenic subjects. Studies applying DXA. [file Image_3.tif]
