# Supplementary material for: Adiponectin and Sarcopenia: A Systematic Review With Meta-Analysis
Source: Front Endocrinol (Lausanne). 2021 Apr 15;12:576619. doi: 10.3389/fendo.2021.576619 (PMC8082154; doi:10.3389/fendo.2021.576619)
Supplement: Supplementary file 5 [file DataSheet_1.pdf]

ID	DOI or PMID	Only_Abstract/ Title	_Fulltext
1	10.1007/s00330-018-5973-2		√
2	5970-7. doi: 10.1210/jc.2005-0261.	√	
3	10.1016/j.mce.2016.02.001	√	
4	10.1016/j.cell.2012.10.050	√	
5	10.1016/j.exger.2013.04.008	√	
6	10.1016/j.obmed.2017.09.005	√	
7	10.1016/j.eurger.2017.07.010	√	
8	0.1016/j.jacc.2008.12.040	√	
9	10.1111/j.1365-2796.2006.01663.x	√	
10	10.1016/j.bbamcr.2016.01.016	√	
11	10.1016/j.bbadis.2015.10.029	√	
12	PMID: 23732375	√	
13	10.1053/j.jrn.2008.02.001	√	
14	10.1016/j.transproceed.2014.09.014	√	
15	10.1016/S1665-2681(19)31790-9	√	
16	10.1016/j.bbmt.2017.11.024	√	
17	10.1253/circj.cj-08-0961	√	
18	10.1016/S1474-4422(14)70085-7	√	
19	10.1016/j.cardiores.2007.02.010	√	
20	10.1016/j.bone.2010.08.010		√
21	10.1016/j.numecd.2008.07.002	√	
22	10.1086/680862	√	
23	10.1007/s00223-010-9376-x		√
24	10.1016/j.ijcard.2011.06.061	√	
25	10.3109/00365513.2012.687759	√	
26	10.1210/en.2012-1765	√	
27	10.1016/j.jstrokecerebrovasdis.2016.10.045	√	
28	10.1038/nutd.2012.18	√	
29	10.1016/j.numecd.2011.03.012	√	
30	0.1016/B978-0-12-398313-8.00011-7	√	
31	10.1111/j.1463-1326.2012.01626.x	√	
32	10.1161/ATVBAHA.110.216804	√	
33	10.1074/jbc.M501149200	√	
34	10.1210/me.2013-1401	√	
35	10.1016/j.rmed.2013.08.016	√	
36	10.1016/j.atherosclerosis.2011.11.038	√	
37	10.2337/diacare.27.3.739	√	
38	10.1152/ajpcell.00030.2008	√	
39	10.1038/oby.2010.296	√	
40	10.1016/j.yjmcc.2015.06.020	√	
41	10.1161/HYPERTENSIONAHA.106.079509	√	

42	10.1016/j.trsl.2006.12.008	√	
43	10.1038/sj.ijo.0802387	√	
44	10.1016/j.orcp.2006.10.002	√	
45	10.2353/ajpath.2010.100363	√	
46	10.1016/j.mehy.2011.04.008	√	
47	10.1016/j.ygyno.2015.02.012	√	
48	10.1016/j.cmet.2013.04.005	√	
49	10.1038/oby.2008.487	√	
50	10.1016/j.cyto.2016.12.018	√	
51	10.1038/s41598-018-37115-3	√	
52	10.1016/j.genrep.2018.11.006	√	
53	10.1002/cphy.c170046	√	
54	10.3109/17477160903111748	√	
55	10.1002/jcsm.12086	√	
56	10.1128/MCB.02279-06	√	
57	10.1016/j.cccn.2005.07.011	√	
58	10.1016/j.cmet.2007.06.003	√	
59	10.2337/db07-0690	√	
60	10.1111/j.1463-1326.2005.00510.x	√	
61	10.3390/ijms18061321	√	
62	10.1016/j.atherosclerosis.2019.10.021	√	
63	10.1016/j.metabol.2014.06.011	√	
64	10.1002/cncr.21690	√	
65	10.1111/j.1365-2265.2008.03242.x	√	
66	10.1016/j.atherosclerosis.2012.09.015	√	
67	10.1016/j.cyto.2016.06.028	√	
68	10.1016/j.diabet.2013.11.004	√	
69	10.1016/j.diabet.2007.08.002	√	
70	10.1007/BF03401534	√	
71	10.1002/ajhb.21087	√	
72	10.1053/j.jrn.2004.09.034	√	
73	10.1093/gerona/glp155		√
74	10.1016/j.exger.2017.10.014	√	
75	10.1016/s1534-5807(02)00199-5	√	
76	10.1016/j.cyto.2012.10.023	√	
77	10.1016/j.coemr.2019.02.003	√	
78	10.1016/j.mad.2013.11.008	√	
79	10.1016/j.metabol.2010.10.005	√	
80	10.1152/ajpendo.00116.2005	√	
81	10.1016/j.ejphar.2008.02.048	√	
82	PMID: 16437024.	√	
83	10.1016/j.bbrc.2013.03.030	√	
84	10.1016/j.gtc.2018.07.016	√	

85	10.1111/j.1872-034X.2011.00833.x	√	
86	10.1016/j.mad.2019.03.003	√	
87	10.1016/j.exger.2012.04.005	√	
88	10.1016/j.mce.2007.07.005	√	
89	10.1016/S2213-8587(14)70034-8	√	
90	10.1016/j.arr.2016.04.003	√	
91	10.1016/j.exger.2008.02.008	√	
92	10.2337/db09-0591	√	
93	10.1371/journal.pone.0186811	√	
94	10.1016/j.exger.2016.02.013	√	
95	10.1681/ASN.2013091025	√	
96	10.1016/j.bbalip.2018.02.006	√	
97	10.1016/j.arr.2019.100935	√	
98	10.1016/j.cmet.2012.01.004	√	
99	10.1016/j.metabol.2004.09.012		√
100	10.1530/JOE-13-0625	√	
101	10.1002/dmrr.321	√	
102	10.1016/j.jcm.2014.07.002	√	
103	10.1016/j.metabol.2012.05.018	√	
104	10.1152/ajpendo.00430.2006	√	
105	10.1016/j.exger.2019.110787	√	
106	10.1016/j.eururo.2008.10.008	√	
107	10.1007/s00125-019-4887-0	√	
108	10.1016/j.maturitas.2016.06.008	√	
109	10.1016/j.cger.2015.04.012	√	
110	10.1016/j.jamda.2017.05.001	√	
111	10.1016/j.molcel.2014.03.005	√	
112	10.1016/j.arr.2016.08.004	√	
113	10.1002/jcsm.12284	√	
114	10.1016/j.pcad.2009.06.004	√	
115	10.1155/2013/601838	√	
116	10.1371/journal.pone.0134648	√	
117	10.1136/bmjdrc-2017-000498	√	
118	10.1016/j.hrtlng.2015.08.006	√	
119	10.2337/db07-1556	√	
120	10.1007/s00394-012-0466-6	√	
121	10.1017/jns.2012.12	√	
122	10.1016/j.cvsm.2011.03.003	√	
123	10.1016/j.ahj.2011.02.014	√	
124	10.1016/j.jsams.2012.03.007	√	
125	10.26402/jpp.2020.1.02	√	
126	10.1016/j.jdiacomp.2015.11.004	√	
127	10.1016/j.anai.2012.07.017	√	

128	10.1007/s00404-015-3656-7	√	
129	10.4082/kjfm.2017.38.1.8	√	
130	10.1016/j.numecd.2016.02.011	√	
131	10.1016/S1607-551X(09)70050-6	√	
132	10.1016/j.joca.2014.11.020	√	
133	10.1016/j.atherosclerosis.2006.11.017	√	
134	10.1016/j.tmsr.2018.09.002	√	
135	10.2337/diabetes.51.7.2325	√	
136	10.1053/j.ajkd.2015.02.325		√
137	10.1016/j.ejim.2013.09.011		√
138	10.1016/j.arcmed.2017.01.003	√	
139	j.atherosclerosis.2008.08.019	√	
140	0.1016/j.diabet.2017.07.001	√	
141	10.1016/j.atherosclerosis.2014.02.033	√	
142	10.1186/1475-2840-10-67.	√	
143	j.atherosclerosis.2009.12.007	√	
144	10.1016/j.cyto.2005.07.008	√	
145	10.1371/journal.pone.0201499	√	
146	10.1371/journal.pone.0225008	√	
147	10.1007/s00198-018-4428-6	√	
148	10.1016/j.jpainsymman.2009.09.021	√	
149	10.3109/15412555.2011.619599	√	
150	10.1159/000328780	√	
151	10.1371/journal.pone.0198969		√
152	10.1111/age.12155	√	
153	10.1016/j.orcp.2018.08.002		√
154	10.3747/pdi.2017.00177	√	
155	10.1016/j.annepidem.2012.07.011	√	
156	10.1016/j.numecd.2010.07.010	√	
157	10.1016/j.orcp.2015.11.008	√	
158	10.1016/j.bone.2011.04.023	√	
159	10.1080/15548627.2019.1628537	√	
160	10.1152/ajpheart.00626.2011	√	
161	10.1016/j.exger.2018.10.006		√
162	10.1152/ajpendo.00002.2006	√	
163	10.1186/s12891-017-1477-5	√	
164	10.1016/j.tifs.2019.03.006	√	
165	10.1016/j.ijcard.2010.04.039	√	
166	10.1016/j.ghir.2009.09.002	√	
167	10.2174/13816128113196660697	√	
168	10.1016/j.cca.2017.07.014	√	
169	10.1016/j.exger.2019.110750	√	
170	10.1016/j.mad.2018.04.003	√	

171	10.1097/GME.0000000000001391	√	
172	10.1016/j.nefroe.2017.04.001		√
173	10.1016/j.jns.2017.08.3250		√
174	10.1210/jc.2011-3123	√	
175	10.1002/jcb.23098	√	
176	10.1530/eje.1.02267	√	
177	10.1016/j.jdiacomp.2018.08.001	√	
178	10.1093/ndt/gfn053	√	
179	10.1016/j.numecd.2014.06.013	√	
180	10.1016/S2213-8587(13)70183-9	√	
181	10.1016/j.jsams.2010.10.587	√	
182	10.1038/sj.ijo.0803716	√	
183	10.1016/j.cmet.2010.08.016	√	
184	10.1002/mnfr.201000176	√	
185	10.1016/j.jbspin.2015.04.010	√	
186	10.1007/s13539-011-0020-z	√	
187	10.1016/j.eurger.2015.06.004		√
188	10.1016/j.cmet.2015.08.022	√	
189	10.1016/j.mam.2011.07.001	√	
190	10.1016/j.exger.2013.10.014	√	
191	10.1016/j.mad.2018.10.005	√	
192	10.1016/j.arr.2009.10.003	√	
193	10.1016/j.febslet.2011.03.015	√	
194	10.1016/j.arr.2020.101038	√	
195	10.1016/j.arr.2016.08.005	√	
196	10.1371/journal.pmed.0040076.	√	
197	0.1016/j.pharep.2016.04.009	√	
198	10.1016/j.critrevonc.2018.05.006	√	
199	10.1016/j.tem.2012.10.006	√	
200	10.1016/j.trecan.2018.10.001	√	
201	10.1016/j.pharmthera.2008.09.009	√	
202	10.1016/j.ijcard.2015.10.115	√	
203	10.1016/j.jacc.2013.01.080	√	
204	10.1016/j.beem.2013.09.001	√	
205	10.1016/j.numecd.2013.08.009	√	
206	10.1016/j.ijcard.2012.09.215	√	
207	10.1016/j.arr.2010.08.008	√	
208	10.1053/j.jrn.2014.10.025	√	
209	10.1016/j.mce.2011.07.011	√	
210	10.3967/bes2017.004	√	
211	10.1159/000087527	√	
212	10.1016/j.pneurobio.2011.10.003	√	
213	10.1210/en.2003-0882	√	

214	10.2337/db10-1106	√	
215	10.1016/j.biochi.2003.10.010	√	
216	10.1210/en.2007-0647	√	
217	10.1016/j.jocd.2019.06.002	√	
218	10.1016/bs.acc.2019.07.012	√	
219	10.1002/hep4.1077	√	
220	0.1016/j.metabol.2015.03.009	√	
221	10.1016/j.lfs.2010.01.003	√	
222	10.1007/s00125-007-0710-4	√	
223	10.1016/j.fct.2010.01.003	√	
224	10.1179/2045772314Y.0000000236	√	
225	10.1152/ajpregu.00142.2016	√	
226	10.1210/en.2006-1371	√	
227	10.1016/j.pnpbp.2007.09.012	√	
228	10.1371/journal.pone.0142319	√	
229	10.1016/j.arr.2013.09.002	√	
230	PMID: 24149611	√	
231	10.1210/jc.2005-2556	√	
232	10.1519/JSC.0b013e31827e1644	√	
233	10.1016/j.metabol.2009.06.032	√	
234	10.1016/j.exger.2004.03.024.	√	
235	10.1002/jcsm.12417		√
236	10.1016/j.metabol.2016.07.002	√	
237	10.1210/jc.2013-2373	√	
238	10.1371/journal.pone.0094463	√	
239	10.1007/s10522-013-9428-5	√	
240	10.1016/j.cophys.2019.03.004	√	
241	Endocrine reviews, 2018, 39(2)	√	
242	10.2337/db19-733-P	√	
243	10.1016/j.atherosclerosis.2018.08.046	√	
244	10.1016/j.atherosclerosis.2014.05.940	√	
245	10.1016/j.archger.2014.02.008	√	
246	10.1016/j.critrevonc.2018.06.011	√	
247	10.1016/j.chest.2016.12.006	√	
248	10.1016/j.joa.2017.07.009	√	
249	10.1016/j.jpeds.2011.05.015	√	
250	10.1016/j.lungcan.2014.01.020	√	
251	10.1080/21623945.2015.1048051	√	
252	10.1210/en.2007-0020	√	
253	10.1093/ije/dyt003	√	
254	10.1016/j.mad.2013.12.001	√	
255	10.1055/s-0030-1267174		√
256	10.1038/oby.2009.232	√	

257	10.1249/MSS.0000000000000658	√	
258	10.1016/j.atherosclerosis.2012.07.013	√	
259	10.1016/j.atherosclerosis.2017.06.031	√	
260	10.1016/j.archger.2015.02.005	√	
261	10.1074/jbc.M311113200	√	
262	10.3389/fphys.2019.00459	√	
263	10.1016/j.bbalip.2016.02.023	√	
264	10.1016/j.gene.2012.04.052	√	
265	10.1016/j.neurobiolaging.2015.02.030	√	
266	10.1210/jc.2009-0436	√	
267	10.1016/j.biocel.2013.07.016	√	
268	10.1016/j.mehy.2016.10.025	√	
269	10.1016/j.nutres.2014.04.009	√	
270	PMID: 23272528	√	
271	10.1016/j.metabol.2005.09.005	√	
272	j.atherosclerosis.2010.05.005	√	
273	10.5603/PiAP.2015.0019	√	
274	10.1007/s00415-017-8405-3	√	
275	10.1016/j.arcmed.2010.11.007	√	
276	10.1016/j.metabol.2013.09.017	√	
277	10.1016/j.exger.2011.11.007		√
278	10.1016/j.ejphar.2009.02.030	√	
279	10.1016/j.nut.2018.03.052	√	
280	10.1016/j.clnu.2009.05.014	√	
281	10.1016/j.arcmed.2011.03.002	√	
282	10.1016/j.metabol.2015.09.009	√	
283	10.1530/EJE-11-0537	√	
284	10.1016/j.transproceed.2018.06.028	√	
285	10.1007/s11357-011-9304-7	√	
286	10.1210/jc.2003-031315	√	
287	10.1016/j.thromres.2003.08.004	√	
288	10.1016/j.cca.2009.04.018	√	
289	10.1016/j.thromres.2016.09.003	√	
290	10.14283/jfa.2017.13	√	
291	10.1152/ajpendo.00620.2012	√	
292	10.1055/s-0035-1564132	√	
293	10.1016/j.bbadis.2014.11.015	√	
294	10.1016/j.metabol.2008.06.017	√	
295	10.1152/ajpendo.00506.2005	√	
296	10.1210/jc.2008-2505	√	
297	10.1097/MCO.0b013e3282f0ecd2	√	
298	10.1016/j.arr.2018.06.005	√	
299	10.3945/jn.108.096362	√	

300	10.1089/jmf.2014.0016	√	
301	10.3389/fnagi.2017.00268	√	
302	10.1016/j.bbagen.2019.129414	√	
303	10.1007/s00508-015-0883-7	√	
304	10.1016/j.jnutbio.2009.01.006	√	
305	10.14814/phy2.13599	√	
306	10.1016/j.metabol.2012.05.003	√	
307	10.1155/2015/534320	√	
308	10.1186/1475-2840-13-47	√	
309	10.1016/j.molmet.2016.08.009	√	
310	10.1210/en.2011-1490	√	
311	10.1038/s41390-019-0428-2	√	
312	10.1016/j.redox.2017.05.004	√	
313	10.1016/j.atherosclerosis.2019.01.029	√	
314	10.1007/978-3-642-17214-4_2	√	
315	10.1242/dev.095810	√	
316	j.drudis.2018.05.009	√	
317	10.1016/j.biocel.2018.08.013	√	
318	10.1093/annonc/mdu085	√	
319	Iranian journal of endocrinology and metabolism, 2010, 12(1)	√	
320	10.1016/j.exger.2017.08.036	√	
321	10.1111/cpf.12005	√	
322	10.1016/j.ejbas.2016.12.002	√	
323	10.1002/hep.25539	√	
324	Iranian journal of endocrinology and metabolism, 2017, 18(5), 368-377	√	
325	10.1007/s11655-016-2518-x	√	
326	10.1371/journal.pone.0191121	√	
327	PMID: 24881626	√	
328	10.1016/j.lfs.2010.03.004	√	
329	10.1016/j.cca.2010.02.069	√	
330	10.1016/j.nut.2014.05.014	√	
331	10.1152/japplphysiol.00683.2005	√	
332	10.1016/j.fertnstert.2008.01.027	√	
333	10.1515/jcim-2013-0053	√	
334	10.1002/dmrr.2662	√	
335	10.1016/j.ejphar.2013.12.036	√	
336	10.1378/chest.08-1705	√	
337	10.1016/j.metabol.2015.12.006	√	
338	FASEB journal, 2017, 31(1)	√	
339	10.1016/j.endinu.2019.09.004	√	
340	10.1002/jbmr.2701	√	

341	10.1539/joh.16-0125-OA	√	
342	10.1080/02640414.2016.1142107	√	
343	10.1111/j.1440-1681.2007.04786.x	√	
344	10.3390/nu10070946	√	
345	10.1016/j.ypmed.2015.09.014	√	
346	10.1139/apnm-2018-0355	√	
347	10.1038/oby.2006.96	√	
348	10.1210/jc.2012-1219	√	
349	10.3390/nu10081070	√	
350	10.1016/j.steroids.2011.06.004	√	
351	10.1200/JCO.2017.75.7526	√	
352	10.2169/internalmedicine.50.4380	√	
353	0.1007/s12576-011-0174-7	√	
354	10.1016/j.ctim.2017.11.023	√	
355	10.1016/j.curtheres.2017.07.002	√	
356	10.1093/ajh/hps050	√	
357	10.1186/s12967-016-1044-0	√	
358	10.5551/jat.2089	√	
359	10.1093/eurheartj/ehu322	√	
360	10.1002/dmrr.1185	√	
361	10.1007/s00125-019-4866-5	√	
362	10.1007/s11655-016-2407-3	√	
363	10.1177/0269881106069637	√	
364	10.1016/j.numecd.2012.07.005	√	
365	10.1016/j.ijcard.2014.11.099	√	
366	10.1111/dom.12112	√	
367	10.12965/jer.1938368.184	√	
368	10.1249/MSS.0000000000001250	√	
369	10.1016/j.clinbiochem.2013.12.019	√	
370	10.1016/j.ghir.2015.12.009	√	
371	1.368.553.820.171.300.000	√	
372	10.1210/en.2013-1166	√	
373	10.1152/ajpendo.00089.2007	√	
374	10.1007/s00421-016-3466-z	√	
375	10.1016/j.phrs.2016.09.029	√	
376	/10.1111/dom.12745	√	
377	10.1016/j.clinthera.2016.01.017	√	
378	ACTRN12618001215213	√	
379	10.1378/chest.07-0227		√
380	10.1136/bmjdrc-2018-000537	√	
381	10.1016/j.metabol.2009.08.015	√	
382	10.1016/j.metabol.2014.10.024	√	
383	10.1016/s1098-3597(08)60026-5	√	

384	10.1016/j.domaniend.2015.11.007	√	
385	10.1152/ajpheart.01032.2009	√	
386	10.5935/abc.20180086	√	
387	10.1016/j.cmet.2012.08.012	√	
388	10.1016/j.biomaterials.2019.119416	√	
389	10.1016/j.atherosclerosis.2013.02.026	√	
390	10.1152/ajpendo.00123.2007	√	
391	10.1053/j.gastro.2003.08.029	√	
392	10.1152/ajpregu.00228.2014	√	
393	10.2353/ajpath.2010.090647	√	
394	10.1016/j.bbrc.2008.02.092	√	
395	10.1016/B978-0-12-800101-1.00005-3	√	
396	10.1371/journal.pone.0173984	√	
397	10.1016/S2213-8587(18)30204-3	√	
398	10.18632/oncotarget.3587	√	
399	10.1016/j.clnu.2018.12.022	√	
400	10.1530/JOE-14-0008	√	
401	10.1677/joe.1.06227	√	
402	10.1152/ajpendo.00707.2009	√	
403	10.1053/j.jrn.2013.01.001	√	
404	10.1016/j.transproceed.2019.04.057	√	
405	10.1074/jbc.M409580200	√	
406	0.1007/s00198-015-3223-x	√	
407	10.1016/j.cger.2015.04.001	√	
408	10.1155/2016/4219735	√	
409	10.1111/dom.12680	√	
410	10.2337/db16-1771-2041	√	
411	10.1152/japplphysiol.00890.2007	√	
412	10.1016/j.maturitas.2017.09.003	√	
413	0.1186/s12986-015-0052-z	√	
414	10.1002/jbmr.5650251305	√	
415	10.1002/jcsm.12166	√	
416	10.1016/j.peptides.2011.01.027	√	
417	10.1249/MSS.0000000000000338	√	
418	10.1111/j.1743-6109.2011.02337.x	√	
419	doi.org/10.1016/j.cct.2015.10.002	√	
420	10.1111/jog.13482	√	
421	10.2527/2005.833565x	√	
422	10.1152/ajpendo.00155.2004	√	
423	10.1111/age.12082	√	
424	10.2169/internalmedicine.48.1102	√	
425	10.1016/j.addr.2014.10.021	√	
426	10.1002/hep.24666	√	

427	10.1016/j.eurger.2016.09.005	√	
428	10.1038/ismej.2017.24	√	
429	10.1038/sj.ijo.0802853	√	
430	10.1210/en.2011-1518	√	
431	PMID: 17598332	√	
432	10.1093/gerona/62.12.1393	√	
433	10.2337/db08-0006	√	
434	10.1016/j.jand.2012.08.013	√	
435	10.1530/EC-12-0003	√	
436	10.1016/B978-0-12-802844-5.00011-7	√	
437	10.1152/ajpregu.00328.2011	√	
438	10.1016/j.metabol.2012.09.002	√	
439	10.1016/j.mam.2016.06.001	√	
440	10.1016/j.arr.2017.01.006	√	
441	10.1053/j.jrn.2017.07.007	√	
442	10.1016/j.mad.2019.03.007	√	
443	10.1053/j.jvca.2018.08.002	√	
444	10.1016/j.ecl.2013.02.006	√	
445	10.1016/j.trre.2016.07.006	√	
446	10.1161/CIRCULATIONAHA.114.015248	√	
447	10.1016/j.nut.2010.08.005	√	
448	10.1016/j.mehy.2014.06.014	√	
449	10.1016/j.jvs.2014.02.045	√	
450	10.1016/j.archger.2017.04.006	√	
451	10.1186/1471-2318-14-8	√	
452	10.1016/j.mce.2015.02.010	√	
453	10.2337/dc07-1257	√	
454	10.1016/j.bbrc.2007.09.128	√	
455	10.1186/1471-2350-13-22	√	
456	10.1016/j.beem.2017.02.005	√	
457	10.1016/j.clnu.2014.08.010	√	
458	10.1152/ajpendo.00460.2007	√	
459	10.1016/j.arr.2018.02.002	√	
460	PMID: 12173692	√	
461	10.2337/db06-1405	√	
462	10.1016/j.bone.2017.08.005		√
463	10.1016/j.cardiores.2007.04.011	√	
464	10.1016/j.tem.2013.12.006	√	
465	10.1152/physiolgenomics.00042.2011	√	
466	10.1186/1743-7075-9-90	√	
467	10.1016/j.nutres.2012.05.001	√	
468	10.1016/j.jff.2019.103679	√	
469	10.1016/j.cyto.2019.01.011	√	

470	10.1016/j.beem.2013.05.003	√	
471	NCT01343641	√	
472	10.1111/j.1440-1681.2007.04786.x	√	
473	10.1016/j.arr.2017.09.001	√	
474	10.1093/ndt/gfq817	√	
475	10.1016/j.exger.2018.11.014	√	
476	10.1155/2015/783296	√	
477	0.1210/jc.2015-1787	√	
478	10.3892/mmr_00000292	√	
479	10.1210/jc.2018-01808	√	
480	10.1016/j.orcp.2011.04.008	√	
481	10.1016/j.ijcard.2016.11.194	√	
482	10.1097/HJR.0b013e32833b09d9	√	
483	10.1016/j.cca.2013.12.037	√	
484	10.1016/j.numecd.2015.03.010		√
485	10.1210/jc.2008-0009	√	
486	10.1155/2012/395757	√	
487	10.1016/j.ijcard.2015.01.057		√
488	10.2337/db07-0932	√	
489	10.1007/s00421-011-2073-2	√	
490	10.1016/j.cger.2019.07.011	√	
491	PMID: 31929469	√	
492	10.1007/s40520-017-0840-6		√
493	10.2310/JIM.0b013e3182746ce8	√	
494	10.1016/j.ultrasmedbio.2019.05.029	√	
495	10.1093/gerona/glv123		√
496	10.1016/j.molmed.2010.03.002	√	
497	10.1007/s00125-016-4046-9	√	
498	PIMID:25489546	√	
499	10.1016/j.jnutbio.2010.01.001	√	
500	10.1007/s12020-018-1550-3	√	
501	10.1111/j.1559-4564.2007.06597.x	√	
502	10.1089/met.2010.0018	√	
503	10.1016/j.eurger.2012.02.008	√	
504	10.1080/19490976.2016.1239680	√	
505	10.1016/j.jpba.2016.03.027		√
506	10.1001/archinte.168.15.1609	√	
507	10.1093/jb/mvt008	√	
508	10.1007/s12041-011-0027-4	√	
509	10.1111/j.1523-1755.2005.00200.x	√	
510	10.1016/j.critrevonc.2019.102857	√	
511	0.1016/j.clnu.2015.06.010	√	
512	NCT04096469	√	

513	10.1210/en.2017-00460.	√	
514	10.1016/j.ekir.2017.12.009	√	
515	10.1016/j.surg.2015.02.002	√	
516	10.1016/j.atherosclerosis.2016.07.897	√	
517	10.1016/j.surg.2015.08.047	√	
518	Iranian journal of endocrinology and metabolism, 2010, 12(3)	√	
519	10.1016/j.metabol.2014.04.004	√	
520	10.1111/jdi.12731	√	
521	10.1016/j.fertnstert.2010.05.018	√	
522	10.1016/j.diabres.2017.11.031	√	
523	10.1016/j.gtc.2019.09.005	√	
524	10.1016/j.thromres.2008.01.010	√	
525	10.1016/j.cyto.2006.09.002	√	
526	10.1089/met.2017.0011	√	
527	10.1016/j.dsx.2015.09.007	√	
528	10.1111/j.1749-6632.2002.tb04292.x	√	
529	10.1016/j.diabres.2006.02.014	√	
530	10.1016/j.clnesp.2019.05.006	√	
531	10.1016/j.bone.2010.12.018	√	
532	10.1016/j.tem.2016.09.005	√	
533	10.1016/j.mad.2016.12.008	√	
534	10.1016/j.arr.2019.100980	√	
535	10.1038/sj.ki.5001969	√	
536	10.1016/j.metabol.2013.07.004	√	
537	10.1016/j.bone.2016.01.019	√	
538	10.1186/s12891-018-2325-y	√	
539	10.1016/j.metabol.2009.12.011	√	
540	10.1007/s40520-018-1029-3		√
541	10.1080/21551197.2018.1564200	√	
542	10.1093/annonc/mdx815	√	
543	10.1053/j.jrn.2012.08.013	√	
544	10.1016/j.cger.2008.03.006	√	
545	10.3899/jrheum.150280		√
546	10.2337/dc08-1994	√	
547	10.1016/j.mce.2012.12.009	√	
548	10.1016/j.peptides.2014.10.006	√	
549	PMID: 21681172	√	
550	10.1016/j.amjcard.2015.07.060	√	
551	10.1016/j.arr.2014.02.007	√	
552	10.1016/j.lfs.2018.10.014	√	
553	10.1016/j.numecd.2013.03.006		√
554	10.1016/j.metabol.2008.05.001	√	

555	10.1016/j.exger.2018.06.013	√	
556	10.1016/j.yclnex.2018.10.004	√	
557	10.1016/j.psyneuen.2018.12.227	√	
558	10.1016/j.yexmp.2013.10.002	√	
559	10.1016/j.bone.2015.07.013	√	
560	10.1016/j.numecd.2018.06.020	√	
561	10.33963/KP.14989	√	
562	10.1016/j.cmet.2019.12.002	√	
563	10.1016/j.numecd.2009.03.002	√	
564	10.1016/j.cger.2014.08.019	√	
565	10.1186/s12944-017-0547-x	√	
566	10.2337/db10-0698	√	
567	10.1016/S1388-1981(02)00342-6	√	
568	10.1172/jci.insight.128405	√	
569	10.2337/db09-1541	√	
570	10.1016/j.arr.2006.03.004	√	
571	10.1038/ncomms12205	√	
572	10.1530/EJE-07-0169	√	
573	10.1530/EJE-11-0289	√	
574	10.1093/gerona/62.3.330	√	
575	10.1016/j.diabet.2016.07.027	√	
576	10.1016/j.ijcard.2007.09.004	√	
577	PMID: 17392141	√	
578	10.1186/s12933-018-0755-3	√	
579	10.1016/j.metabol.2008.03.019	√	
580	10.1089/neu.2018.6136	√	
581	10.1016/j.clgc.2013.05.004	√	
582	10.1016/j.ecl.2015.07.010	√	
583	10.1016/j.disamonth.2019.06.008	√	
584	doi.org/10.1016/j.dld.2019.06.002	√	
585	10.1016/j.ajg.2018.08.003	√	
586	10.1016/j.mad.2015.03.006	√	
587	10.1038/s41598-017-12597-9	√	
588	10.1002/mnfr.201900072	√	
589	10.1016/j.maturitas.2013.03.014	√	
590	10.2337/diabetes.53.6.1621	√	
591	10.3945/ajcn.115.109173	√	
592	10.1007/s00421-014-2879-9	√	
593	10.1002/oby.20212	√	
594	10.1016/j.juro.2012.11.017	√	
595	10.1016/j.metabol.2011.10.005	√	
596	10.1016/j.cell.2016.07.031	√	
597	10.1016/j.diabet.2012.03.001	√	

598	j.1463-1326.2011.01429.x	√	
599	10.3109/08037051.2013.791411	√	
600	doi.org/10.1016/j.jacc.2014.04.083	√	
601	10.1136/thoraxjnl-2017-211073	√	
602	10.1016/j.dsx.2007.11.008	√	
603	10.1016/j.phrs.2018.01.009	√	
604	10.1016/j.ijcard.2011.09.079	√	
605	10.1371/journal.pone.0051357	√	
606	10.1016/j.jss.2013.06.002	√	
607	10.1210/en.2003-0870	√	
608	10.1016/j.bbalip.2017.05.011	√	
609	10.1016/j.biotechadv.2012.09.005	√	
610	10.1016/j.mad.2016.12.005	√	
611	10.1007/s10545-011-9338-0	√	
612	10.1016/j.bbcan.2018.07.008	√	
613	10.1016/j.biocel.2013.06.024	√	
614	10.1016/j.mad.2019.04.003	√	
615	doi.org/10.1016/j.arr.2008.07.002	√	
616	10.1111/j.1749-6632.1999.tb07793.x	√	
617	10.1210/jc.2018-01771	√	
618	10.1016/j.cca.2006.07.005	√	
619	10.1016/j.arr.2016.09.012	√	
620	10.1016/j.arr.2011.09.001	√	
621	10.1089/scd.2016.0166	√	
622	10.1016/j.metabol.2015.05.008	√	
623	10.1161/01.CIR.0000150391.38660.9B	√	
624	10.1016/j.arr.2015.03.002	√	
625	10.3899/jrheum.170513	√	
626	10.1016/j.bone.2016.10.024	√	
627	10.1016/j.jhep.2018.06.005	√	
628	10.1016/j.amjcard.2014.05.050	√	
629	10.1038/srep41792	√	
630	10.1186/s13098-019-0458-2. P	√	
631	10.1016/j.imlet.2018.09.002	√	
632	10.1016/j.cyto.2017.12.008	√	
633	10.1210/en.2005-0362	√	
634	10.1016/j.dld.2019.03.002	√	
635	10.1016/j.drudis.2012.02.001	√	
636	10.1016/j.archger.2013.06.001		√
637	10.1371/journal.pone.0166804	√	
638	10.1016/j.arr.2018.05.006	√	
639	10.1016/j.cmet.2017.11.002	√	
640	10.1677/JOE-10-0104	√	

641	10.1016/j.mce.2015.05.033	√	
642	10.1371/journal.pone.0178123		√
643	10.1016/j.phrs.2015.12.010	√	
644	10.1210/en.2011-1343	√	
645	10.1016/j.maturitas.2019.03.016	√	
646	10.1038/ijo.2010.123	√	
647	10.1161/CIRCRESAHA.111.258673	√	
648	10.1016/j.bbamcr.2018.02.010	√	
649	10.1016/j.jceh.2017.11.001	√	
650	10.1016/j.mad.2014.01.008	√	
651	10.1016/j.arr.2017.02.002	√	
652	10.1016/j.cardfail.2019.03.007	√	
653	10.1016/B978-0-12-801238-3.00154-9	√	
654	10.1016/j.metabol.2018.11.001	√	
655	10.1016/j.metabol.2018.10.011	√	
656	10.1016/j.exger.2013.06.007	√	
657	10.1016/j.metabol.2018.11.014	√	
658	10.1016/j.pan.2019.08.008	√	
659	10.1016/B978-0-12-809657-4.10899-3	√	
660	10.1016/j.cgh.2018.07.006	√	
661	10.1016/j.beem.2013.04.01	√	
662	10.1016/j.dld.2017.07.004	√	
663	10.1053/j.jrn.2013.09.002	√	
664	10.1016/j.metabol.2013.05.019	√	
665	10.1159/000489691	√	
666	doi.org/10.1016/j.jtho.2017.07.022	√	
667	10.1172/JCI31021	√	
668	10.1096/fj.08-125674	√	
669	10.1038/ijo.2010.135	√	
670	10.1016/j.suc.2016.03.013	√	
671	10.1016/j.smrv.2018.08.009	√	
672	10.1016/j.pathophys.2017.08.003	√	
673	10.2337/dc10-1002	√	
674	10.1016/j.clnu.2011.05.009	√	
675	10.1074/jbc.C200312200	√	
676	10.2337/db12-1042	√	
677	10.1016/j.rbmo.2009.05.003	√	
678	10.1016/j.bone.2012.08.117	√	
679	10.1016/j.cger.2008.03.012	√	
680	10.1016/j.rehab.2016.04.002	√	
681	10.1016/j.exger.2020.110884		√
682	10.2147/IJICMR.S19007	√	
683	10.1016/j.lfs.2009.02.026	√	

684	10.2337/db14-1611	√	
685	10.1093/humrep/dew279	√	
686	10.1017/S0954422412000145	√	
687	10.1159/000074501	√	
688	10.1590/s0100-879x2012007500097	√	
689	10.1016/j.cca.2008.10.009	√	
690	10.1007/s10068-018-0378-1	√	
691	10.1038/ijo.2008.233	√	
692	10.1016/j.cophys.2018.02.013	√	
693	10.1016/j.cger.2011.03.005	√	
694	10.1371/journal.pone.0122660	√	
695	10.1016/j.numecd.2009.10.007	√	
696	10.2337/diacare.28.3.755	√	
697	10.1016/j.metabol.2007.06.014		√
698	10.1016/j.numecd.2013.04.015		√
699	10.1016/j.numecd.2010.01.014	√	
700	10.1016/j.cca.2006.01.021		√
701	10.1210/jc.2004-2549	√	
702	10.1016/j.jacc.2006.05.054	√	
703	10.1016/j.jstrokecerebrovasdis.2008.10.001	√	
704	10.1038/s41393-018-0089-8	√	
705	10.1055/s-2005-865661	√	
706	10.1080/10790268.2017.1405154	√	
707	10.1111/j.1600-0838.2006.00602.x	√	
708	10.1016/j.ahj.2011.09.029	√	
709	10.2147/DMSO.S220050	√	
710	10.1538/expanim.62.347	√	
711	10.2527/jas.2009-2597	√	
712	10.1016/j.yhbeh.2010.02.008	√	
713	10.1371/journal.pone.0011307	√	
714	10.1016/j.clnu.2018.01.032	√	
715	10.1016/j.nut.2015.01.009	√	
716	10.1016/j.yapd.2016.04.005	√	
717	10.1016/j.healun.2018.07.015	√	
718	10.1038/ijo.2013.77	√	
719	10.1016/j.mad.2013.12.006	√	
720	10.1253/circj.cj-11-1549	√	
721	10.1016/j.diabres.2016.07.017	√	
722	10.1016/j.plipres.2015.03.001	√	
723	/j.atherosclerosis.2015.02.009	√	
724	10.1371/journal.pone.0210120	√	
725	10.1016/j.clnu.2016.06.007	√	
726	10.1073/pnas.041591798	√	

727	10.18632/aging.102044	√	
728	10.1056/NEJMoa1113966	√	
729	10.1017/S0007114513002584	√	
730	10.1053/j.ajkd.2018.06.024	√	
731	10.1016/j.kjms.2017.05.008	√	
732	10.1016/j.diabres.2018.02.009	√	
733	10.1016/j.afos.2015.07.009	√	
734	10.1016/j.redox.2015.06.019	√	
735	10.1093/jn/135.11.2517	√	
736	10.1016/j.ajpath.2014.05.012	√	
737	10.1002/pbc.25601	√	
738	10.1152/ajpgi.00526.2007	√	
739	10.1007/s00125-005-1686-6	√	
740	10.1152/ajpendo.00245.2006	√	
741	10.1016/j.beem.2013.06.003	√	
742	10.1016/j.plipres.2013.12.001	√	
743	10.1016/j.mce.2016.11.014	√	
744	10.1016/j.mad.2018.07.004	√	
745	10.5099/aj090200133	√	
746	10.3177/jnsv.59.393	√	
747	10.1016/j.diabres.2010.09.008	√	
748	10.1016/j.amjcard.2010.01.368	√	
749	10.1016/j.amjcard.2012.12.043	√	
750	10.1016/j.cca.2006.01.022	√	
751	10.1016/j.spinee.2014.06.009	√	
752	10.1152/physiolgenomics.00058.2013	√	
753	10.1159/000148648	√	
754	10.1097/MAJ.0b013e3182112bcd	√	
755	10.1016/j.dsx.2017.09.004	√	
756	10.2147/DMSO.S16175	√	
757	10.1016/j.maturitas.2012.09.009	√	
758	10.12659/MSM.889362	√	
759	10.1016/j.cyto.2012.10.021	√	
760	10.1016/j.exger.2015.07.006		√
761	10.2337/db07-1739	√	
762	10.1152/japplphysiol.00485.2013	√	
763	10.1249/MSS.0b013e3182644984	√	
764	10.1016/j.regpep.2014.10.001	√	
765	10.1016/j.mad.2010.02.007	√	
766	10.1677/jme.1.00010	√	
767	10.1073/pnas.1205675109	√	
768	10.1371/journal.pone.0014205	√	
769	10.1016/j.clinthera.2019.01.006	√	

770	10.1074/jbc.M807120200	√	
771	10.1016/j.dsx.2016.12.027	√	
772	10.1053/j.gastro.2017.01.046	√	
773	10.1016/j.mad.2019.01.008	√	
774	10.1053/j.jrn.2007.05.006		√
775	10.1074/jbc.M309469200	√	
776	0.1016/j.diabet.2019.02.006	√	
777	10.3390/biom9120780	√	
778	10.1016/j.eujim.2018.09.002	√	
779	10.1016/j.diabres.2018.05.041	√	
780	10.1016/j.bbagen.2009.01.008	√	
781	10.1111/j.1464-5491.2009.02868.x	√	
782	10.1016/j.pathophys.2018.05.001	√	
783	10.1016/j.peptides.2013.11.003	√	
784	10.1016/j.arr.2015.05.001	√	
785	10.1016/j.jhep.2015.05.014	√	
786	10.1016/j.jvs.2014.10.094	√	
787	doi.org/10.1016/j.ijcard.2017.03.155	√	
788	10.1016/j.jhep.2015.02.031	√	
789	10.1016/j.clnu.2016.03.028		√
790	10.1155/2013/204164	√	
791	10.1016/j.arr.2016.09.008	√	
792	10.1016/j.diabres.2016.03.019	√	
793	10.1515/jpem.2009.22.5.409	√	
794	10.1007/s00395-013-0390-9	√	
795	0.1515/pjvs-2016-0056	√	
796	10.1016/j.amepre.2011.09.023		√
797	10.1111/j.1532-5415.2011.03865.x		√
798	10.2337/db10-0650	√	
799	10.1007/s11897-019-00437-y	√	
800	10.1016/j.diabres.2013.05.014		√
801	10.1016/j.dsx.2009.07.012	√	
802	10.3390/ijerph120911528	√	
803	10.1016/j.nut.2016.06.014		√
804	10.1016/j.cyto.2009.01.003	√	
805	10.1016/j.jad.2011.07.029	√	
806	10.1016/j.dsx.2019.04.048	√	
807	10.1016/j.arcmed.2010.08.012	√	
808	10.1016/j.metabol.2005.08.011	√	
809	10.1016/j.exger.2019.03.005		√
810	10.1016/j.metabol.2010.05.001	√	
811	10.1016/j.metabol.2006.04.015	√	
812	10.1016/j.ejcdt.2014.01.004	√	

813	10.1016/j.euf.2015.06.012	√	
814	10.1016/j.exger.2009.10.012	√	
815	10.1016/j.ejim.2013.02.012		√
816	10.1016/j.metabol.2006.12.008		√
817	10.1016/j.aller.2016.09.004	√	
818	10.1007/s40520-016-0626-2		√
819	10.1016/j.bone.2013.07.018	√	
820	10.3892/mmr.2013.1522	√	
821	10.1210/jc.2007-0325	√	
822	10.1186/cc13048	√	
823	10.1016/j.trsl.2009.08.003	√	
824	10.1016/j.diabres.2016.11.005	√	
825	10.5665/sleep.4568	√	
826	10.1111/cen.13563	√	
827	10.2337/diacare.27.2.308	√	
828	10.1113/expphysiol.2012.070300	√	
829	10.1152/ajpheart.00593.2012	√	
830	10.1111/j.1365-2265.2008.03373.x	√	
831	10.1016/j.arr.2018.08.001	√	
832	10.1111/j.1463-1326.2008.00856.x	√	
833	10.1016/j.aprim.2019.07.019	√	
834	10.1016/j.arr.2013.05.007	√	
835	10.1016/j.diabres.2003.12.008	√	
836	10.1016/j.jada.2011.03.011	√	
837	10.2337/db13-1423	√	
838	10.1055/s-0030-1253404		√
839	10.1016/j.metabol.2010.10.001	√	
840	10.1016/S0006-291X(02)00680-0	√	
841	10.3390/nu11071461	√	
842	10.1016/j.dsx.2020.06.006	√	
843	10.1055/s-0030-1269905	√	
844	10.14814/phy2.13848	√	
845	10.1016/j.jacl.2020.01.002		√
846	10.1016/B978-0-12-809657-4.99647-9	√	
847	10.1371/journal.pone.0154119	√	
848	10.1055/s-0030-1269846	√	
849	10.1016/j.archger.2016.04.017	√	
850	10.1016/j.mce.2016.09.013	√	
851	10.1007/s00421-014-2953-3	√	
852	10.1016/j.atherosclerosis.2009.01.022	√	
853	10.3390/ijms19051339	√	
854	10.3390/molecules23051205	√	
855	10.1007/s12020-017-1460-9	√	

856	10.1038/s41598-017-16979-x	√	
857	10.1210/jc.2008-0782	√	
858	10.1249/MSS.0000000000000526	√	
859	10.7150/ijms.4.19	√	
860	10.1016/j.ijcard.2013.10.031	√	
861	10.1111/j.1748-1716.2012.02409.x	√	
862	10.1074/jbc.M207198200	√	
863	JPRN-UMIN000020427	√	
864	10.1016/j.fertnstert.2007.03.085	√	
865	10.1210/jc.2004-0224	√	
866	10.1016/j.prp.2016.02.005	√	
867	10.1016/j.cmet.2017.09.020	√	
868	10.1093/gerona/glz001		√
869	10.1016/j.metabol.2019.03.003	√	
870	10.1073/pnas.0403382101	√	
871	10.1016/j.amjhyper.2006.12.010	√	
872	10.1016/j.bcp.2019.02.030	√	
873	10.1016/j.fct.2019.05.052	√	
874	PMID: 28673384	√	
875	10.1139/apnm-2015-0123	√	
876	10.1016/j.tem.2006.10.010	√	
877	10.1016/j.jacc.2016.06.072	√	
878	10.1093/gerona/gly017		√
879	10.1016/j.arr.2012.03.002	√	
880	10.1016/j.jevs.2018.11.005	√	
881	10.1530/JOE-17-0656	√	
882	10.1016/j.metabol.2008.01.031	√	
883	10.1002/dmrr.2926	√	
884	10.1016/j.diabres.2009.02.002	√	
885	10.1016/j.resinv.2015.05.005	√	
886	10.1093/ckj/sfs176	√	
887	10.1159/000489691	√	
888	10.1007/s00198-010-1370-7.	√	
889	10.1016/j.scispo.2011.11.004	√	
890	0.1007/s00125-007-0699-8	√	
891	10.5717/jenb.2015.19.1.19	√	
892	10.22088/jbums.17.4.7	√	
893	10.1016/j.exger.2019.03.007	√	
894	10.1111/j.1464-5491.2009.02897.x	√	
895	10.1016/j.neubiorev.2017.04.030	√	
896	10.1016/j.gene.2018.05.040	√	
897	10.1016/j.dsx.2019.01.040	√	
898	10.1242/jeb.163840	√	

899	10.1186/1745-6215-15-431	√	
900	10.1158/1055-9965.EPI-17-0766	√	
901	10.1016/j.sbspro.2012.05.138	√	
902	10.1111/j.1365-2265.2006.02474.x	√	
903	PMID: 24431310		√
904	10.1016/j.ddmod.2019.03.002	√	
905	10.1016/j.obmed.2016.12.002	√	
906	10.1016/j.hfc.2019.08.008	√	
907	10.1139/apnm-2014-0256	√	
908	10.1016/j.regpep.2011.11.004		√
909	10.1016/j.smim.2018.09.003	√	
910	10.12968/hmed.2016.77.6.362	√	
911	10.1194/jlr.M800610-JLR200	√	
912	10.1016/j.cgh.2017.04.004	√	
913	10.1074/jbc.M103241200	√	
914	10.1016/j.juro.2014.12.086	√	
915	10.2337/dc06-0145	√	
916	10.1016/j.mce.2008.07.002	√	
917	10.1016/j.atherosclerosis.2015.03.012	√	
918	10.1016/j.cyto.2012.02.005	√	
919	10.1007/s11033-008-9260-1	√	
920	10.1016/j.ekir.2017.01.009	√	
921	10.1016/j.jbspin.2018.12.005	√	
922	10.1002/lsm.22395	√	
923	10.1016/j.regpep.2011.04.003		√
924	10.1016/j.humimm.2015.10.008	√	
925	10.1016/j.ajp.2019.04.006	√	
926	10.1016/j.ijpp.2018.04.001	√	
927	10.1111/j.1748-1716.2005.01502.x	√	
928	10.2174/0929867325666180713154505	√	
929	10.1164/rccm.201603-0446OC	√	
930	10.1016/S0047-6374(01)00287-1	√	
931	10.1016/j.joca.2019.08.003	√	
932	10.1016/j.chemphyslip.2019.05.005	√	
933	10.1002/hep.27395	√	
934	10.1016/j.niox.2019.02.006	√	
935	10.1073/pnas.0705408104	√	
936	10.1016/j.cmet.2017.02.006	√	
937	10.1016/j.cyto.2017.12.008	√	
938	10.1530/eje.0.1480293	√	
939	10.1016/j.ocarto.2019.100019	√	
940	10.1016/j.trsl.2013.07.011	√	
941	10.1016/j.exger.2009.04.001	√	

942	10.1016/j.jcyt.2017.07.002	√	
943	10.1002/mnfr.201300285	√	
944	10.1016/j.jnutbio.2011.04.001	√	
945	10.1016/j.ekir.2017.12.010	√	
946	10.1007/s00125-007-0867-x	√	
947	10.1002/jcsm.12129	√	
948	10.1152/ajpendo.00169.2005	√	
949	10.1093/gerona/gls077		√
950	10.1016/j.clnu.2016.11.007	√	
951	10.1016/j.arr.2018.07.004	√	
952	10.1530/JOE-16-0363	√	
953	10.1016/j.bbrc.2005.09.044	√	
954	10.3390/ijms151120022	√	
955	10.1016/S2213-8587(15)00283-1	√	
956	10.1016/j.metabol.2009.08.020	√	
957	10.1016/j.archger.2019.03.021	√	
958	10.1016/j.ejrad.2016.04.005	√	
959	0.1152/physiolgenomics.00226.2007	√	
960	10.1016/j.bone.2014.03.008	√	
961	10.1038/ijo.2008.141	√	
962	10.1016/j.ijcard.2016.04.036	√	
963	10.1093/ndt/gfl552	√	
964	10.1016/j.bcp.2019.08.023	√	
965	10.1016/j.cca.2018.05.046	√	
966	10.14814/phy2.12556	√	
967	10.1016/j.rmed.2017.08.007	√	
968	10.1016/j.jim.2010.09.012	√	
969	10.1016/j.sjbs.2017.11.008	√	
970	10.1152/ajpheart.00394.2012	√	
971	10.1016/j.atherosclerosis.2008.11.007		√
972	10.1089/met.2008.0035	√	
973	10.1016/j.diabres.2011.12.014	√	
974	10.3945/jn.109.119388	√	
975	10.1152/japplphysiol.00862	√	
976	10.1016/j.clnu.2006.02.008	√	
977	0.1016/j.arr.2013.11.002	√	
978	10.1016/j.cell.2013.12.012	√	
979	10.14814/phy2.14062	√	
980	10.1016/j.semnephrol.2008.10.002	√	
981	10.1016/j.bbrc.2019.06.141	√	
982	10.1039/c8fo00898a	√	
